# Defining, quantifying, and reporting intensity, dose, and dosage of neurorehabilitative interventions focusing on motor outcomes

**DOI:** 10.3389/fresc.2023.1139251

**Published:** 2023-08-10

**Authors:** Gaizka Goikoetxea-Sotelo, Hubertus J. A. van Hedel

**Affiliations:** ^1^ Swiss Children’s Rehab, University Children’s Hospital Zurich, University of Zurich, Affoltern am Albis, Switzerland; ^2^ Children’s Research Center, University Children’s Hospital Zurich, University of Zurich, Zurich, Switzerland; ^3^ Department of Health Sciences and Technology, ETH Zurich, Zurich, Switzerland

**Keywords:** dose-response relationship, TIDieR, stroke, cerebral palsy, rehabilitation, intensity, dose, dosage

## Abstract

**Introduction:**

Determining the minimal amount of therapy needed for positive neurorehabilitative outcomes is important for optimizing active treatment interventions to improve motor outcomes. However, there are various challenges when quantifying these relationships: first, several consensuses on the definition and usage of the terms intensity, dose, and dosage of motor interventions have been proposed, but there seems to be no agreement, and the terms are still used inconsistently. Second, randomized controlled trials frequently underreport items relevant to determining the intensity, dose, and dosage of the interventions. Third, there is no universal measure to quantify therapy intensity accurately. This “perspectives” paper aims to increase awareness of these topics among neurorehabilitation specialists.

**Defining, quantifying, and reporting:**

We searched the literature for definitions of intensity, dose, and dosage and adapted the ones we considered the most appropriate to fit the needs of neurorehabilitative interventions. Furthermore, we suggest refining the template for intervention description and replication (TIDieR) to enhance the reporting of randomized controlled trials. Finally, we performed a systematic literature search to provide a list of intensity measures and complemented these with some novel candidate measures.

**Discussion:**

The proposed definitions of intensity, dose, and dosage could improve the communication between neurorehabilitation specialists and the reporting of dose and dosage in interventional studies. Quantifying intensity is necessary to improve our understanding of the minimal intensity, dose, and dosage of therapy needed to improve motor outcomes in neurorehabilitation. We consider the lack of appropriate intensity measures a significant gap in knowledge requiring future research.

## 1. Introduction

The development of several active neurorehabilitative interventions to improve motor outcomes has been fueled by comprehending the complex mechanisms underlying activity-dependent motor recovery. The field agrees that exercises should be goal-directed and repeated multiple times to facilitate neural adaptations and improve therapeutic outcomes (1, 2). Although several interventions have proven their effectiveness in different neurological diagnoses, literature shows that not each patient with the same diagnosis responds similarly to such interventions (3, 4). Furthermore, similar interventions do not always show equivalent results within the same patient group (see, for example, strength training, physical activity, or hippotherapy) (5, 6). The field is working on identifying factors that could explain these conflicting results. For example, identifying patient characteristics and biomarkers predictive of improved motor outcomes can be meaningful to personalize a therapeutic application and select potential responders. Furthermore, by identifying the dose-response relationship of an active neurorehabilitative intervention, one can determine the minimal amount of treatment needed to improve motor outcomes.

Research to improve the personalized application of active therapeutic interventions and optimize dose-response relationships is important. However, we noticed several issues that could be hindering breakthroughs in this field. First, we noticed a need for more consensus on defining and using the terms intensity, dose, and dosage. This is important, as agreeing on the terminology provides a framework and common language for researchers and clinicians to improve research and reporting. Second, randomized controlled trials (RCTs) often do not adequately report items relevant for estimating the intensity, dose, and dosage of the intervention. Better reporting should facilitate study replication and refine analyses such as dose-response relationships. Third, proper and valid intensity measures would enable accurate quantification of what the patient actually does during therapy, which is relevant when calculating dose-response relationships. However, there is no universal measure to quantify therapy intensity accurately.

The general aim of this “perspectives” paper is to increase awareness of these topics among neurorehabilitation specialists. We will focus on the intensity, dose, and dosage of active neurorehabilitative interventions targeting motor outcomes, as the terminology might differ in other areas, e.g., language or cognitive domains [e.g., see (7)].

## 2. Definitions

The *first issue* we noted is the disagreement on the usage of intensity, dose, and dosage in the literature. Particularly intensity has been used very diversely. Intensity has been referred to as the “frequency of repetitions of the desired movement” (8–10), “amount of external work” (11), or “amount of time that is dedicated to practice” (12). Intensity has been equated with dose, i.e., the number of hours spent in exercise therapy (13, 14). It has also been referred to as the number of repetitions, training sessions, therapy duration, and patient activity during each of the repetitions (15). A research summit on the proceedings on dosing in children with an injured brain or cerebral palsy concluded that intensity “refers to how hard the child, i.e., the patient, works within the intervention session and is recorded as the number of repetitions per minute, day, or week or amount of work” (16). While we agree primarily with this definition, we deem the definition proposed by Page and other colleagues of the ACRM Stroke Movement Interventions Subcommittee more accurate (17). They refer to intensity as “the amount of physical or mental work put forth by the client during a particular movement or series of movements, exercise, or activity during a defined period of time”. We prefer this definition because it also includes mental work. Additionally, we would delimit the period of time to a single therapy session because, according to the general adaptation syndrome and periodization theories (18, 19), long-lasting changes in the system should result from repeating sessions of sufficient, but not too high, intensity over time. Therefore, we define *intensity* as: *the amount of physical or mental work put forth by the patient during a particular movement or series of movements, exercise, or activity during a therapy session*.

Also the use of dose and dosage must be clarified. Page and colleagues conceptualize dosing “to encompass the total amount of activity performed during the training period” (17) and consider duration as “the length of time during which a single intervention is administered” but also as “the total amount of time that an intervention period occurs”. They do not define dosage but mention dosing schedules and specify frequency as “how often during the program period therapy is provided”.

The previously mentioned research summit (16) and Gannotti and colleagues (20, 21) adapted the concepts from the American College of Sports Medicine guidelines, which proposed the definitions concerning continuous recommendations to remain physically healthy (22), to fit with the vocabulary used in neurorehabilitation. They define dose based on its parameters, including: (1) frequency, or the number of sessions a week and number of weeks, (2) intensity, or how strenuous the exercise is each session, (3) time, or the amount of time per session, and (4) type, or the type of exercise that is performed. Others refer to type as delivery of treatment (17, 23).

The American Medical Association assigns dose and dosage of pharmacological interventions specific meanings (24). Dose is a specified amount of medication taken at one time, usually expressed in milligrams or milligrams per kilogram bodyweight or, for example, the number of drops or pills. Dosage defines how to take the prescribed medication over time: it comprises how many times a day or week a patient should take the dose (frequency) and for how many weeks or months (duration of intervention).

In analogy with the pharmacological definitions, and to be able to differentiate between the outcomes of interventions of equal intensity and dose but different distributions over time (i.e., dosage, e.g., a 2-week 4-hour-a-day program versus a 4-week 2-hour-a-day; see, for example, (25, 26)), we propose the following definitions of dose and dosage for the field of neurorehabilitation: (1) *Dose* should include (i) *the intensity*, and (ii) *the length of the intervention session*. Information on the (2) *dosage,* i.e., the distribution of the therapy, is provided by (i) *the frequency*, i.e., the number of sessions per week, and (ii) *the length of intervention*, i.e., the total number of weeks.

Differently to (16, 20, 21), we did not include the contents, i.e., type of therapy in the definition of dose. We think we should treat this as a separate factor, because to claim the superiority of one therapy type over another, we should match the dose and dosage of the therapies, while keeping the type of therapy as an independent variable.

Additionally, we propose the term “*total dose*”, equaling the total amount of therapy. This would be calculated by *combining dose and dosage*. The total dose is often reported as the total amount of time spent in therapy (hours or minutes). Most reviews report the total dose or differences in the total dose to determine thresholds for improvement or dose-response relationships. For example, “children with cerebral palsy need to practice at least 14–25 h for improving individual goals and 30–40 h for ameliorating general upper limb function” (27) or “16 h of additional therapy within the first six months after stroke result in a small but favorable effect on activities of daily life” (14). In line with our reasoning, it is evident that the actual dose and total dose are difficult to report as they should consider the intensity.

## 3. Reporting

The *second issue* we noted is that most RCTs incompletely describe intensity-, dose-, and dosage-related items of their experimental and control interventions. This low reporting quality could jeopardize identifying the optimal dose-response relationships. Already in 2013, Hoffmann and colleagues brought this topic to the spotlight (28) and developed the Template for Intervention Description and Replication (TIDieR) (29) checklist to help authors better report their interventions. It contains 12 items. Items 3–9 are considered core items essential to replicate an intervention. Items 8–12 are crucial for estimating the truly delivered intensity, dose, and dosage: Item 8. “when and how much” the intervention was delivered. Item 9. “tailoring”, i.e., if the intervention was planned to be personalized or adapted. Item 10. “modifications”, i.e., if, what, how, and when the intervention was modified during the study, and items 11. “how well: planned” and 12. “how well: actual”, which include if the adherence and fidelity to therapy were planned to be assessed and if they actually were evaluated respectively.

The reviews from Hoffmann et al. (30), McEwen et al. (31), Small et al. (32), Monelly et al. (33), and Sakzewski et al. (34) reported on the quality of the items of neurorehabilitative interventions. Proper reporting of item 8 ranged between 31% and 100%; item 9, between 17% and 77%; item 10, between 1% and 10%; item 11, between 11% and 26%; item 12, between 8% and 94%.

Dose-response calculations are usually based on item 8, “when and how much”, which comprises the planned intensity, dose, and dosage, and item 12, “how well actual”, which includes the actually performed intensity, dose, and dosage. These two items showed the highest reporting variability. This variability could be related to the previously discussed problems with defining intensity. Compared to the other reviews, reporting quality was best in the study of McEwen and colleagues (31). They analyzed the reporting of circuit class exercises, a type of cardiorespiratory training with well-defined intensity measures. Therefore, we suggest that items 8 and 12 of the TIDieR checklist could be specified by including the definitions of intensity, dose, dosage, and total dose. Despite the challenges in finding appropriate intensity measures for many specific interventions, these definitions could provide a better base for reporting “how much”.

Another aspect that needs more attention is reporting additional therapies that might contribute to overall functional improvement. Patients often receive other treatments outside the investigated therapy sessions. For example, while the focus might be on comparing an interventional (e.g., robotic training) and control intervention (e.g., conventional physiotherapy), other therapies (e.g., sports therapy, occupational therapy, and rehabilitation nursing) could contribute to the overall improvements in functional outcome. In addition, leisure activities performed outside the therapeutic program, such as going for a stroll with family members or playing with friends, might contain rehabilitative elements contributing to the functional outcomes. While an RCT would control for equality of additional therapies between the interventional and control group, the randomization could still not correct for an underestimated amount of the total dose. Therefore, researchers should consider and report the intensity, dose, and dosage of concomitant therapies and leisure activities to refine calculations of dose-response relationships.

## 4. Quantifying and measuring intensity

**Figure 1:**
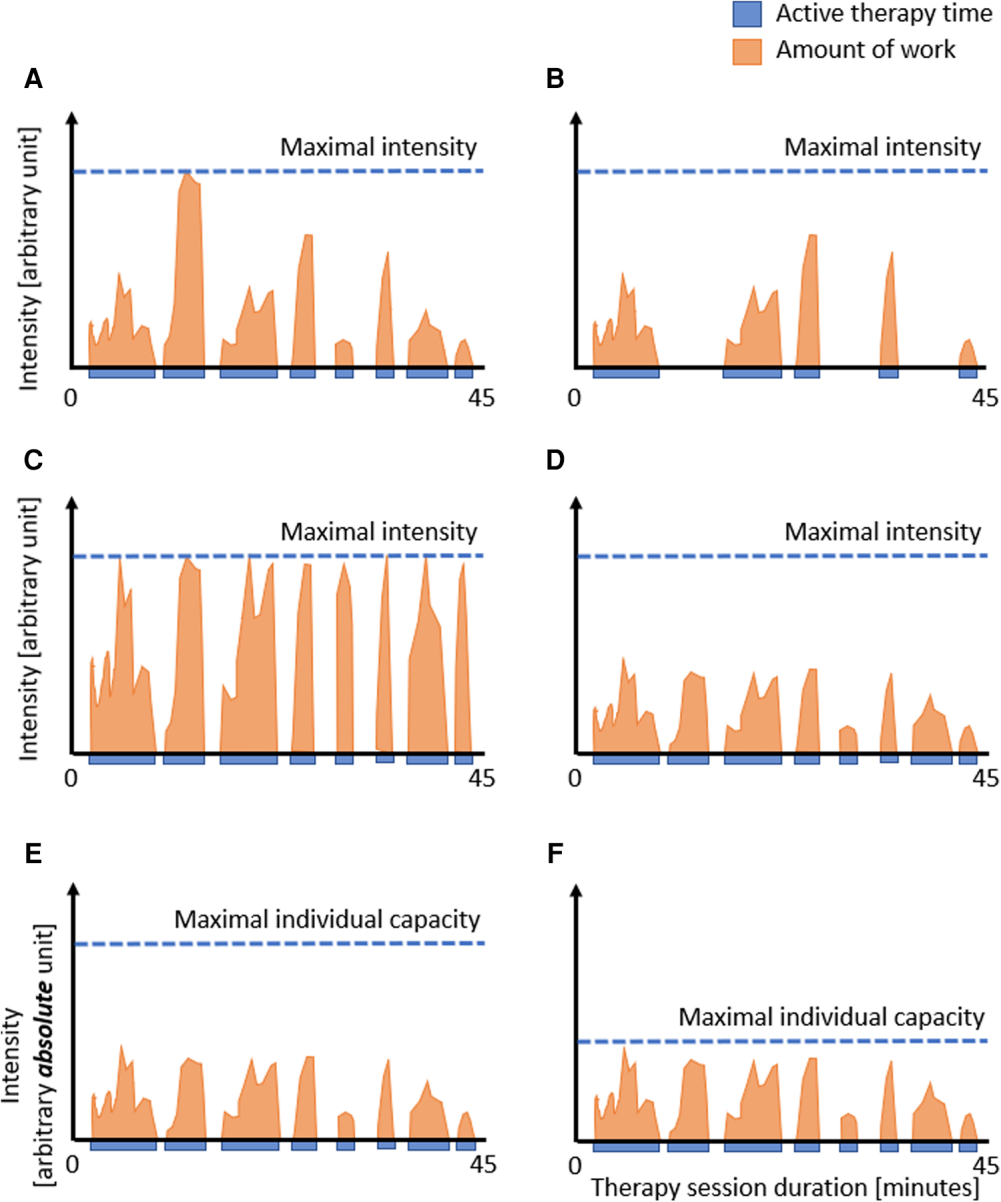
Dose: therapy duration, active therapy time, and intensity. Representation of hypothetical therapy sessions showing the duration of the session, the time the patient is active (blue), and the amount of work the patient performs, i.e., intensity (orange). The figure shows that reporting the therapy session duration (i.e., 45 min.) does not adequately reflect the actual work the patient is doing. Comparing A to B: reporting the active time rather than the duration of the therapy session might improve the estimation of the actual dose. However, even with similar active times (C vs. D), the actual dose can differ, showing the need to include the intensity in such calculations. In the case intensity measures are used that are based on absolute numbers (E and F), such as the number of movement repetitions per minute, steps per session, or heart rate, it is difficult to interpret the levels of intensity between individual patients, as the intensity should also take into account the patient's capacities. In this example, the similar amount of active time and work is not so intensive for the patient in E but highly intensive for the patient in F, reflecting the challenges when absolute measures are not scaled to the capacities of the individual patient.

We can assess the distribution of the intervention during the intervention period (i.e., dosage) and the duration of the therapy program well. As most studies equal intensity and dose, they quantify both with “time spent in therapy”. However, although time spent in therapy seems to be the universal quantification of the amount of treatment, it has been considered a very rough estimate of the actual active contribution of a patient (see also Figure 1) (13, 35).

One suggestion to slightly improve the estimation of the dose is to report the time when the patient is active. However, as visualized in Figures 1A and B, even the active time does not approximate the amount of work the patient does as long as information on the intensity is missing (compare Figures 1C,D). Based on our clinical observations, we can confirm that no two patients perform the same amount of work during a therapy session. This variability may be more prominent in our field, i.e., pediatric neurorehabilitation, where we treat infants, children, and adolescents of various ages with different diagnoses, severity grades, and developmental stages. However, this variability also applies to adult neurorehabilitation. Still, even within the same patient, the amount of work can vary substantially from session to session, for example, due to fatigue, motivation, compliance, daytime, or day of the week. Consequently, we need to assess the intensity; therefore, the *third issue* is how to quantify intensity.

We performed a systematic search to identify which intensity measures have been reported in systematic reviews published in English that investigated active neurorehabilitative interventions aiming to improve motor functions. We performed the search with the following terms in Pubmed on 20.06.2023: ((assess*) OR (quantif*) OR (measur*)) AND (intensity) AND (neurorehabilitation) AND (motor) AND (review). Two researchers (GGS and HvH) independently evaluated each title and abstract to determine whether it fulfilled the inclusion criteria. In case of disagreement, they discussed until a consensus was found. Full-text papers were searched for measures of intensity using the search function (“intensit”).

The search resulted in 76 publications. Agreement between the raters was high (four papers were shortly discussed). The flow diagram is shown in Supplementary Figure S1. We present the results from the 18 remaining papers in Table 1.

**Table 1:** Intensity measures.

Category of measures	Intensity measure	References^a^
Dose- and dosage-related	- Minutes per day - Duration of session - Frequency per week - Duration of the intervention period - Time spent in therapy - Mean hours delivered - Active minutes per session - Dose	Webster et al. (36) Chiu et al. (37) Aramaki et al. (38) Valentín-Gudiol et al. (39) Vloothuis et al. (40) Veerbeek et al. (41) Sehatzadeh (42) Rabadi (43) Medical Advisory Secretariat (44) Cooke et al. (45)
Cardiorespiratory capacity	- (Absolute) heart rate - % heart rate reserve - % heart rate maximum / % peak heart rate - % VO2 maximum - % peak oxygen uptake - % heart rate predicted (e.g., based on age)	Penna et al. (46) Clos et al. (47) Hornby et al. (48) Wiener et al. (49) Hasan et al. (50)
Energy cost	- Metabolic Equivalent of Task (MET)	Wiener et al. (49) Lamotte et al. (51)
Muscle work Muscular capacity	- % of peak power output - Power output or rate of work - Load lifted - % of 1 repetition maximum - % of maximum workload	Clos et al. (47) Hornby et al. (48) Wiener et al. (49)
Movement-related	- Number of repetitions - Repetitions per minute - Movements per minute - Steps per session - Acceleration of upper limb movements	Doumen et al. (52) Hornby et al. (48) Aramaki et al. (38) Lo et al. (53) Veerbeek et al. (41)
Task-related	- Treadmill speed - Treadmill inclination - Walking velocity - Fastest possible speed (over-ground)/maximum tolerated speed - Walking as far as possible with minimal rests - % tolerated speed - Adding weights during walking	Chiu et al. (37) Hornby et al. (48) Wiener et al. (49) Valentín-Gudiol et al. (39) Hasan et al. (50)
Perceived Effort		Penna et al. (46) Clos et al. (47) Hasan et al. (50)

^a^ Multiple referrals possible. The studies included patients with stroke (*n* = 12), broader neurological diagnoses (*n* = 2), multiple sclerosis (*n* = 1), Parkinson's Disease (*n* = 1), cerebral palsy (*n* = 1), and children at risk of neuromotor delay (*n* = 1). The interventions included broad physiotherapeutic and other exercises (*n* = 6), cardiorespiratory interventions (*n* = 4), robotics (*n* = 3) and virtual reality (*n* = 2), treadmill (*n* = 1), constrained-induced movement therapy (*n* = 1), and caregiver-mediated exercises (*n* = 1).

The first observation is that many studies use dose- and dosage-related measures to quantify intensity. As discussed, these items are important, but we do not think that these items quantify intensity.

Second, there are two fields within neurorehabilitation where the intensity can be quantified relatively well. Cardiorespiratory programs target heart rate levels (e.g., at a certain percentage of the maximum heart rate) to provide optimal intensity during therapy. The heart rate is generally easy to assess, even if there are some challenges in patients with autonomic nervous system problems (54). Furthermore, strength training interventions report on the intensity by providing the number of bouts and repetitions complemented with weights or resistances reflecting a certain percentage of the maximal capacity (e.g., at the percentage of the one-repetition-maximum).

Third, movement- and task-related measures can quantify intensity. Observing the number of repetitions is straightforward but time-consuming. Novel technologies like wearable inertial measurement units or rehabilitation therapy technologies could quantify the number of repetitions per session. For example, rhythmical, repetitive movements (e.g., the number of steps) or movements with a well-identifiable onset and end [e.g. (55)] might be relatively easy to assess. However, quantifying the number of complex non-cyclic movements [e.g., during exergaming, see (56)] might be difficult. There are additional challenges when using technologies to quantify intensity. Particularly when patients need considerable support, it remains difficult to differentiate between the work provided by the powered technology and the patient. For some technologies, we instruct patients to “move with the device” or “be as active as possible”. Newer technologies use control strategies where the patient has increased kinematical freedom requiring increased active control of the movements (57). However, these technologies can also not differentiate between the contribution of the device and the patient.

There might be an additional limitation when using these well-quantifiable movement-based or task-based *absolute* intensity measures. The number of movement repetitions per session does not consider the patient's capacity. We visualized this in Figures 1E and F. Shown are two patients who perform at the same “absolute intensity level” (please note the different notation of the y-axis compared to A–D). However, due to the lower capacity of the patient in Figure 1F, one can consider that this patient performs at a much higher *relative* intensity level than the patient displayed in Figures 1E.

Indeed, reporting intensity relative to the capacity of the individual patient lies at the basis of the perceived effort scales that were also reported in the reviews (see Table 1). A well-known example of such a measure is the Borg scale (58). It provides the relative intensity perceived by the patient, seems versatile, and therapy independent. One drawback is that perceived effort scales tend to lose precision in patients with cognitive impairment.

### 4.1 Further considerations on assessing intensity

Assessing the intensity turns out to be very complex and further considerations make it even more challenging. First, an optimal quantitative intensity measure should consider the individual therapeutic goal. For example, in the case of upper limb neurorehabilitation, one goal might be to use the affected hand more often in bimanual tasks, while another could be to improve unilateral hand function. Each goal might require another therapeutic approach and, therefore, a specific intensity measure.

Second, neurorehabilitation is not a pure sensorimotor process; mental processes also play an essential role (59, 60). Therapists can intensify the therapy by increasing the motor component (e.g., more or faster movements) but also the difficulty of the tasks (e.g., increasing the mental load by having the patient practice a task that requires more motor planning). Consequently, intensity measures should quantify the amount of physical work and simultaneously reflect the mental effort the patient needs to undergo to participate in therapy successfully. For example, rather than using a Borg scale, a questionnaire like the NASA Task Load Index might prove useful as it measures workload across various fields, including effort and physical and mental demand (61).

Third, we might need to search for additional measures that quantify intensity, are practical and easy to assess, and take the patient's capacities into account. Candidate measures could be physiological measures like heart rate variability or skin conductance. Studies indicate that heart rate variability or skin conductance can assess changes in cognitive load (62, 63) and react to increments in task difficulty during exercises requiring both motor and mental work, such as video gaming (64), driving (65), or playing chess (66). Such physiological measures might provide an intensity estimation covering all kinds of therapies. Moreover, one could record these signals online, providing immediate biofeedback enabling monitoring of the patient's intensity level, and adjusting the workload throughout the session.

Other physiological intensity measures could assess the level of brain activation in the region of interest. For example, Holper and colleagues (67) analyzed changes in the activation of the primary motor cortex (M1) using functional near-infrared spectroscopy (fNIRS) in healthy adults performing hand exercises of different complexity. We recently investigated whether the prefrontal cortex and the supplementary motor area showed increased activations when children with gait impairments walked in the over-ground bodyweight supporting device Andago compared to treadmill walking (68). Walking in the Andago appears closer to reality and requires more concentration than walking on a treadmill (69). Both studies indicate that the more complex tasks resulted in increased levels of brain activation. However, fNIRS currently seems too complicated to apply clinically (68).

Finally, future studies should investigate the psychometric properties, i.e., the validity, reliability, and responsiveness as well as the practicability to apply such measures under regular clinical conditions.

## 5. Limitations and summary

This perspectives article aims to increase awareness of the meaning and relevance of intensity, dose, and dosage among neurorehabilitation specialists. It has several limitations. We did not systematically search for definitions of intensity, dose, and dosage due to the wide use of these terms in the literature. Our systematic search for intensity measures was limited to one database, and the search terms should be extended. However, we aimed to raise awareness for this topic rather than provide comprehensive overviews of definitions and measures. Furthermore, we clarified throughout the manuscript where the content reflects the authors' opinions.

We present definitions of intensity, dose, and dosage based on the literature, some slightly adjusted to fit the needs of the field of neurorehabilitation. A common language should be the starting point for discussing and further investigating this topic. We identified several issues that can be improved immediately, for example, reporting the active time, therapy frequency, and duration of the overall intervention. Even if the reporting of these items improved, the field needs to work on measures quantifying the intensity. In the long term, we need appropriate intensity measures to adequately report intensity, dose, dosage, and total dose to make informed decisions about the effectiveness of specific therapies. This would allow us to refine our analyses and optimize neurorehabilitative programs by accurately determining dose-response relationships or the minimal total dose needed to improve functional outcomes.

## Data Availability

The original contributions presented in the study are included in the article/**Supplementary Material**, further inquiries can be directed to the corresponding author.
