# Supplementary material for: Defining, quantifying, and reporting intensity, dose, and dosage of neurorehabilitative interventions focusing on motor outcomes
Source: Front Rehabil Sci. 2023 Aug 10;4:1139251. doi: 10.3389/fresc.2023.1139251 (PMC10457006; doi:10.3389/fresc.2023.1139251)
Supplement: Supplementary file 1 [file Datasheet1.pdf]

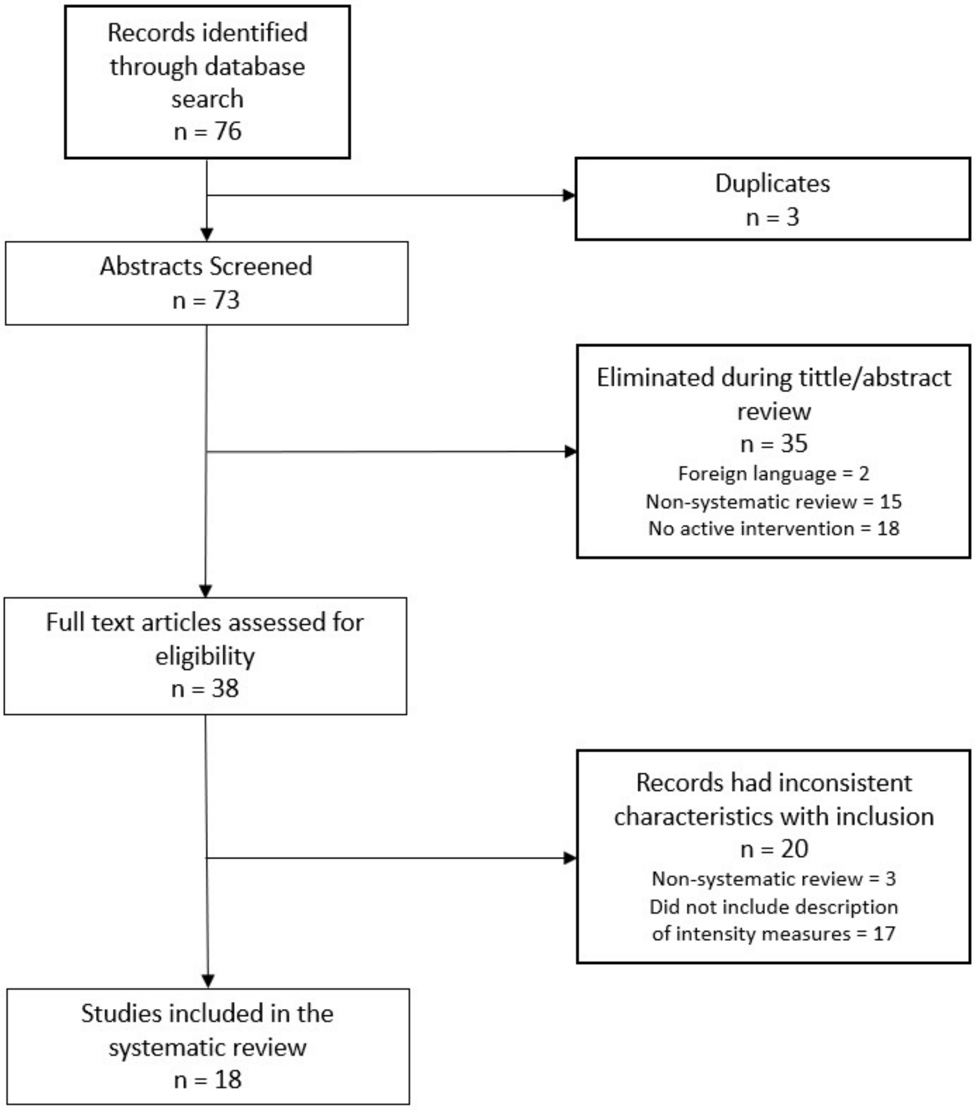


*Supplementary Fig.1. Flow-chart.*

Exclusion of publications was based on the following reasons: duplicates (n=3), not in the English language (n=2), no systematic review (n=15), and not focusing on an active motor intervention (n=18). We evaluated the full-texts of 38 papers. We excluded three more papers because they were not systematic reviews and, consecutively, 17 papers because they did not include quantitative intensity measures. Finally included were 18 papers.
